# The Quad 2.0 Technique: A Single Rectus Femoris Tendon Autograft Solution for Combined Anterior Cruciate Ligament and Double-Bundle Anterolateral Ligament Reconstruction

**DOI:** 10.1016/j.eats.2025.103669

**Published:** 2025-06-02

**Authors:** Victor Sonnery-Cottet, Ali Alayane, Dany Mouarbes, Regis Pailhe, Etienne Cavaignac

**Affiliations:** a Clinique Universitaire du Sport, Centre Hospitalier Universitaire de Toulouse (CHU), Toulouse, France; b Centre Hospitalier de Perpignan (CHP), Perpignan, France; c Cabinet Arthropole, Bayonne, France

## Abstract

Rectus femoris tendon autograft is gaining popularity for anterior cruciate ligament (ACL) reconstruction as the result of advancements in harvesting techniques that minimize soft-tissue dissection and reduce donor-site morbidity. In this Technical Note, we describe a surgical technique for ACL and anterolateral ligament reconstruction in revision ACL cases using a single rectus femoris autograft, with a focus on the harvesting technique to ensure adequate graft length and diameter. Notably, anterolateral ligament fixation is performed without the need for additional femoral or tibial tunnels.

Anterior cruciate ligament (ACL) surgery has significantly evolved over the past 10 years, with growing interest in associated lateral extra-articular procedures. This combination of ACL reconstruction and lateral extra-articular procedures has shown a significant improvement in ACL graft rupture rates without an increase in complications.^1^^,^^2^ The most commonly described surgical techniques include combining the bone–patellar tendon–bone with the iliotibial band (the modified Lemaire procedure), as well as using hamstring tendon grafts for ACL and anterolateral ligament (ALL) reconstruction.^3^, ^4^, ^5^ Many surgical techniques have been described for ACL reconstruction using the rectus femoris (RF) tendon autograft.^6^^,^^7^ In addition, various methods have been used to simplify the harvesting of the RF tendon graft, ensuring sufficient graft size and length while preserving the integrity of the extensor mechanism.^8^^,^^9^ In this Technical Note, we describe an arthroscopic technique for ACL and ALL reconstruction using the RF autograft. This technique focuses on the tips and tricks for harvesting the RF tendon (Video 1), ensuring adequate graft length and size to perform a combined anatomic ACL and double-bundle ALL reconstruction in revision ACL surgeries. Surgical tips and tricks are described in Table 1. Advantages and disadvantages are described in Table 2.

**Table 1:** Surgical Steps, Pearls, and Pitfalls of the Quad 2.0 Technique for Combined Anatomical Anterior Cruciate Ligament (ACL) and Anterolateral Ligament (ALL) Reconstruction Using an RF Tendon Autograft

Surgical Steps	Pearls	Pitfalls
RF graft harvesting	Good dissection of the subcutaneous tissues and identification of the quadriceps tendon. The plane between the RF tendon and the vastus intermedius should be identified at 3 to 4 cm proximal to the superior patellar border and the RF tendon should be dissected starting from the patella and extending up to 8 cm in length before harvesting. RF harvesting should be performed at 20° of knee flexion.	Parallel incisions in the RF tendon should be made more laterally to avoid the vastus medialis muscle. Separation of the superficial layer of the quadriceps tendon from the deep layer before harvesting to avoid penetration into the knee joint.
Femoral tunnel entry point identification and ALL tibial fixation preparation	Small skin markings are made just proximal to the lateral epicondyle before performing the incision to ensure identification of the femoral tunnel entry point, which is located proximal and posterior to the lateral epicondyle.	Avoid dissection distal to the lateral epicondyle after the opening of the fascia lata to prevent injury of the lateral collateral ligament (LCL). The anchor should be inserted in the same direction of the drilled lateral tibial plateau tunnel to avoid anchor breakage.
Femoral and tibial tunnel creation	Drilling the tibial and the femoral tunnels with a 4.5-mm diameter reamer allows correction of tunnels direction using the guidewire before definitive drilling with the second 9-mm diameter reamer.	Two-stage drilling of the tibial tunnel is performed to avoid tibial eminence fracture.
RF graft preparation	A 20-cm graft length is necessary to prepare the RF graft, which is divided into 2 parts: the intra-articular portion for ACL reconstruction and the extra-articular portion for ALL graft reconstruction.	The graft diameter should be assessed before the creation of the tibial and femoral tunnels to prevent mismatch between the tunnels and graft diameter.
RF graft passage and fixation	A soft k-wire should be passed through the femoral tunnel before graft passage from the femur to the tibia to simplify femoral fixation with the interference screw. The 2 bundles of the ALL should be passed under the facia lata for anatomical ALL reconstruction.	The interference screw prominence from the femoral tunnel can induce pain related to facia lata friction. ALL fixation should be performed in full knee extension and neutral rotation to avoid nonisometric ALL reconstruction.

RF, rectus.

**Table 2:** Advantages and Disadvantages of the Surgical Technique

Advantages	Disadvantages
The RF graft offers a strong and sufficiently long graft for combined ACL and ALL reconstruction. Preventing the use of allograft in ACL revision surgeries and its associated risks. Anatomical ACL and double-bundle ALL reconstruction with a lower ACL graft failure rate. Effective solution for multiligament knee injuries, preventing hamstring tendon harvesting and ensuring optimal knee valgus stability. ALL reconstruction is performed without the need for additional femoral or tibial tunnels, avoiding the risk of tunnels conversion. Avoid complications of other revision ACL techniques (hematoma, anterior knee pain, patellar fracture).	RF tendon harvesting requires a moderate learning curve. Risk of quadriceps tendon weakness post operatively. Risk of capsular injury during graft harvesting. Risk of screw irritation with iliotibial band with femoral fixation.

ACL, anterior cruciate ligament; ALL, anterolateral ligament; RF, rectus.

## Surgical Technique

### Patient Setup

Under general anesthesia, the patient is placed in a supine position with a tourniquet applied to the operative thigh. The foot is positioned on padded supports, allowing the knee to be evaluated for its full range of motion, with stability confirmed at 90° flexion.

Preoperative evaluation includes the Lachman test to confirm anterior knee instability and the pivot shift test to assess anterolateral rotatory instability. In addition, previous skin incisions are identified and marked before the beginning of the revision procedure.

### RF Graft Harvesting

**Fig 1:**
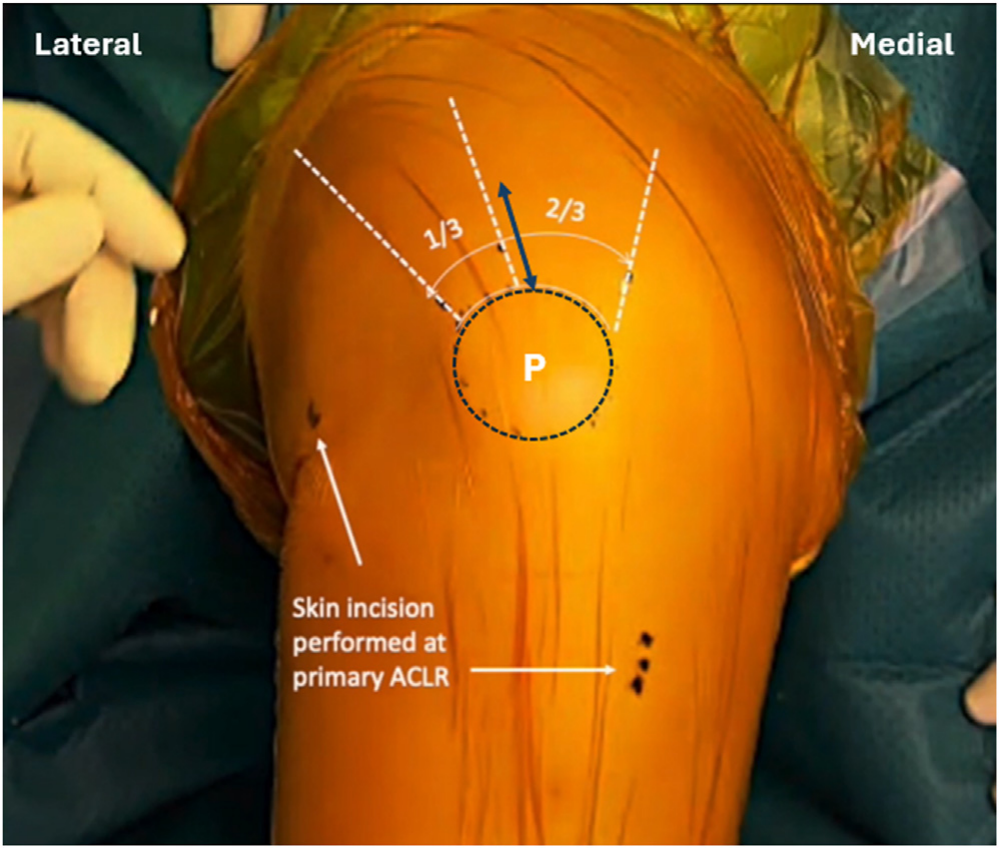
Intraoperative photograph of the left knee showing the vertical skin incision (arrow), which begins at the upper border of the patella, one third of the distance from the lateral patellar corner. (P, patella.)

A 4-cm vertical skin incision is created, starting from the upper border of the patella at one third of the distance from the lateral patellar corner (Fig 1). The subcutaneous tissues are dissected to expose the quadriceps tendon, which is then carefully separated from the surrounding subcutaneous tissues.

**Fig 2:**
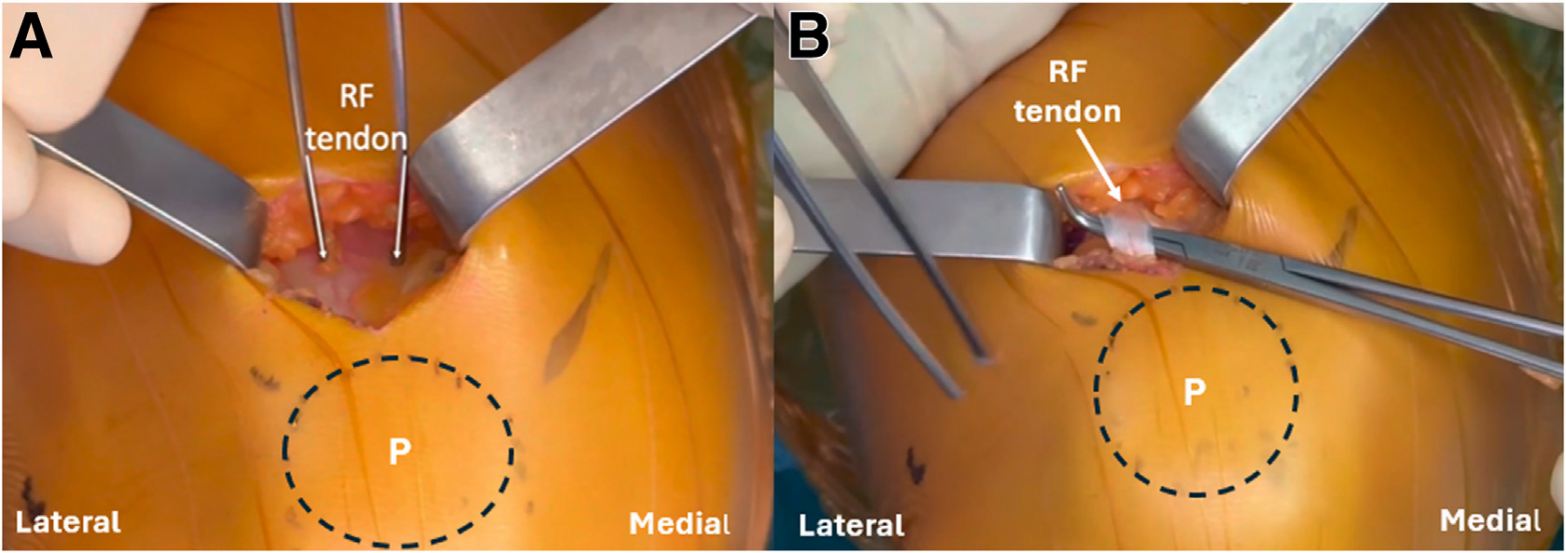
Intraoperative photograph of the left knee showing the rectus femoris identification after dissection of the subcutaneous tissues (A). Two parallel incisions, 1 cm apart, are made, and the superficial layer of the rectus femoris tendon is separated from the deep layer (B). (P, patella.)

**Fig 3:**
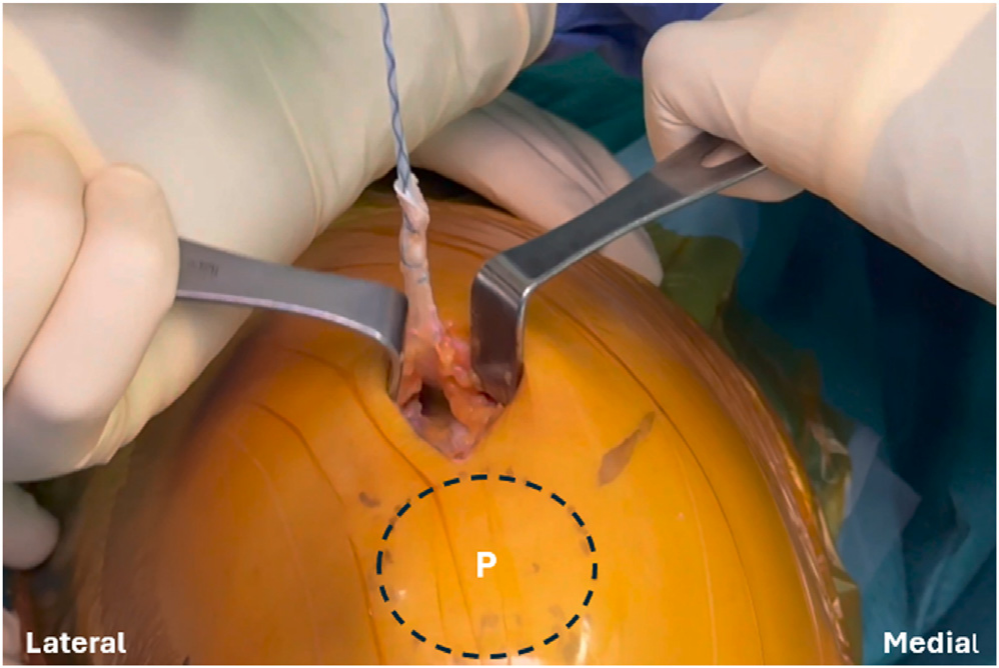
Intraoperative photograph of the left knee showing the detachment of the superficial layer of the rectus femoris tendon from the upper border of the patella. It is then secured with No. 2 nonabsorbable sutures. (P, patella.)

Two parallel incisions, 1 cm apart, are made in the RF tendon to separate its superficial layer from the deep layer (Fig 2). The dissection to separate the 2 quadriceps tendon layers is more easily performed 3 to 4 cm proximal to the upper border of the patella. The superficial layer is then carefully detached from the upper border of the patella and secured with 2 nonabsorbable sutures (Fig 3).

**Fig 4:**
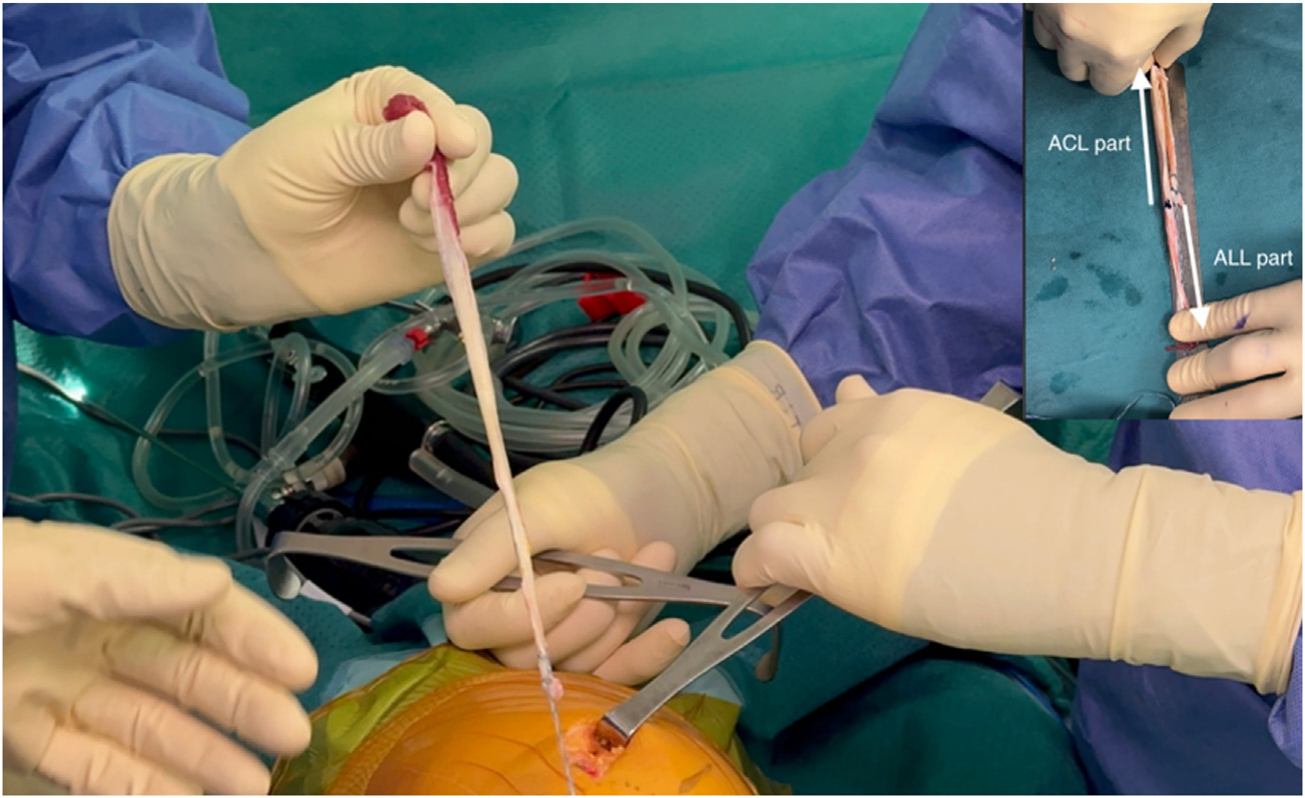
Intraoperative photograph of the left knee showing the harvested superficial layer of the rectus femoris tendon. The graft is then folded in half to accurately determine the required tunnel diameter for the anterior cruciate ligament part.

The superficial RF tendon is meticulously dissected from the other quadriceps tendons and then harvested using a closed tendon stripper (AR-1279L; Arthrex). The harvested RF tendon is then folded in half to accurately assess the required tunnel diameter (Fig 4).

### Femoral Tunnel Entry Identification and ALL Tibial Fixation Preparation

**Fig 5:**
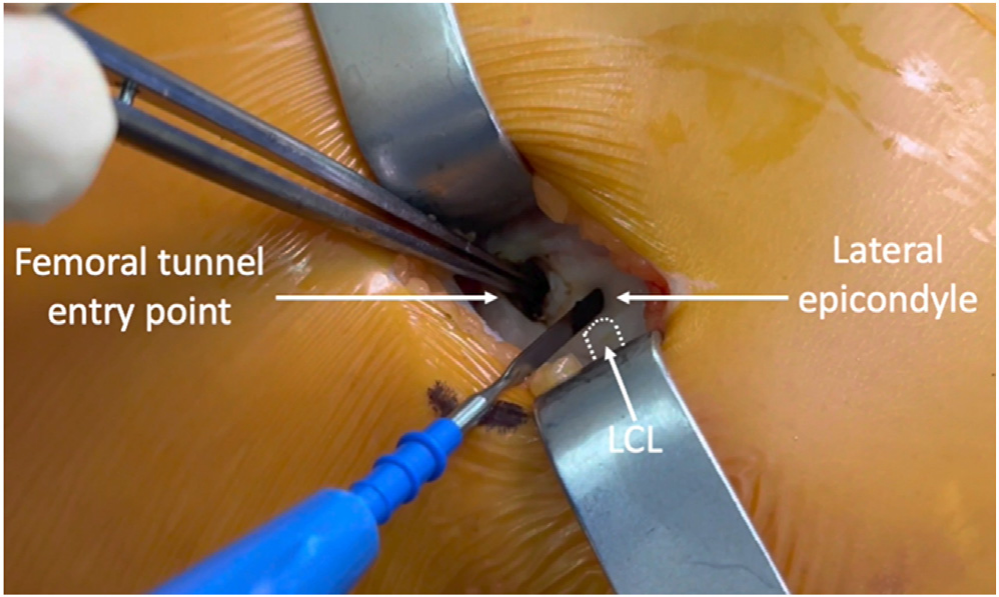
Intraoperative photograph of the left knee showing a 2-cm skin incision performed proximal to the lateral epicondyle. The lateral collateral ligament is identified and protected, and the femoral tunnel entry point is identified just proximal to the lateral epicondyle.

**Fig 6:**
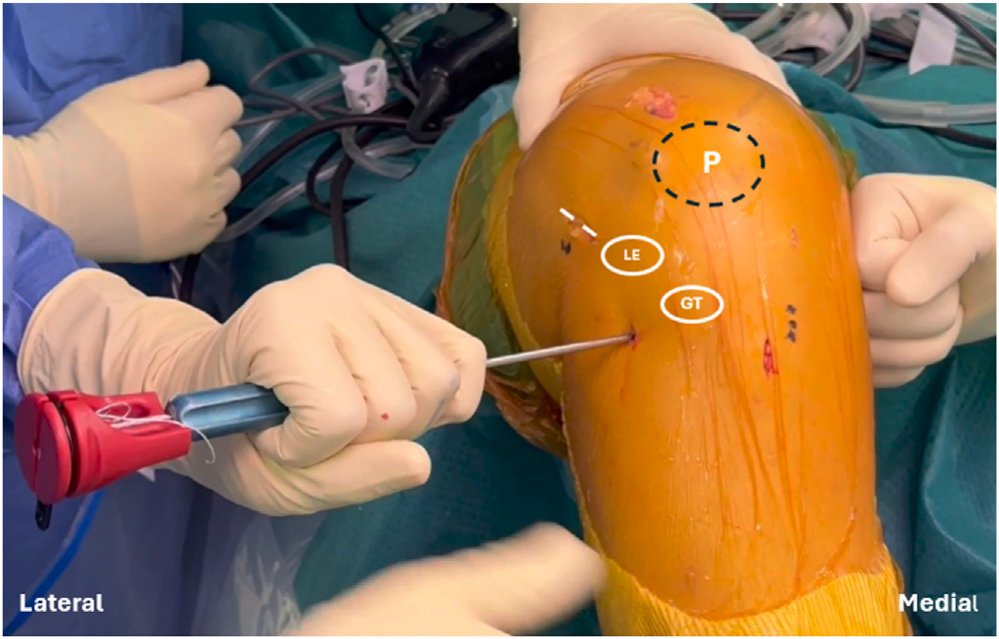
Intraoperative photograph of the left knee showing the tibial FiberTak insertion for anterolateral ligament reconstruction, located posterior to the Gerdy tubercule. (GT, Gerdy tubercle; LE, lateral epicondyle; P, patella.)

A 2-cm skin incision is performed just proximal to the lateral epicondyle. The iliotibial band is then incised to expose and identify the femoral tunnel entry point, located immediately proximal to the lateral epicondyle (Fig 5). A 1.8-mm knotless FiberTak Soft Anchor (Arthrex) is inserted into the lateral cortex and positioned slightly posterior to the Gerdy tubercle and approximately 1 cm distal to the articular surface (Fig 6).

### Femoral and Tibial Tunnel Creation

An ACL tibial tunnel is created using a standard outside-in technique. A guidewire is inserted through the anterior tibial cortex, and it’s overdrilled sequentially using 4.5-mm and 9-mm diameter reamers. For the ACL femoral tunnel, an outside-in guide is used to insert a pin through the previously marked entry point (Fig 5) and then overdrilled with 4.5-mm and 9-mm diameter reamers, respectively.

### RF Graft Preparation

**Fig 7:**
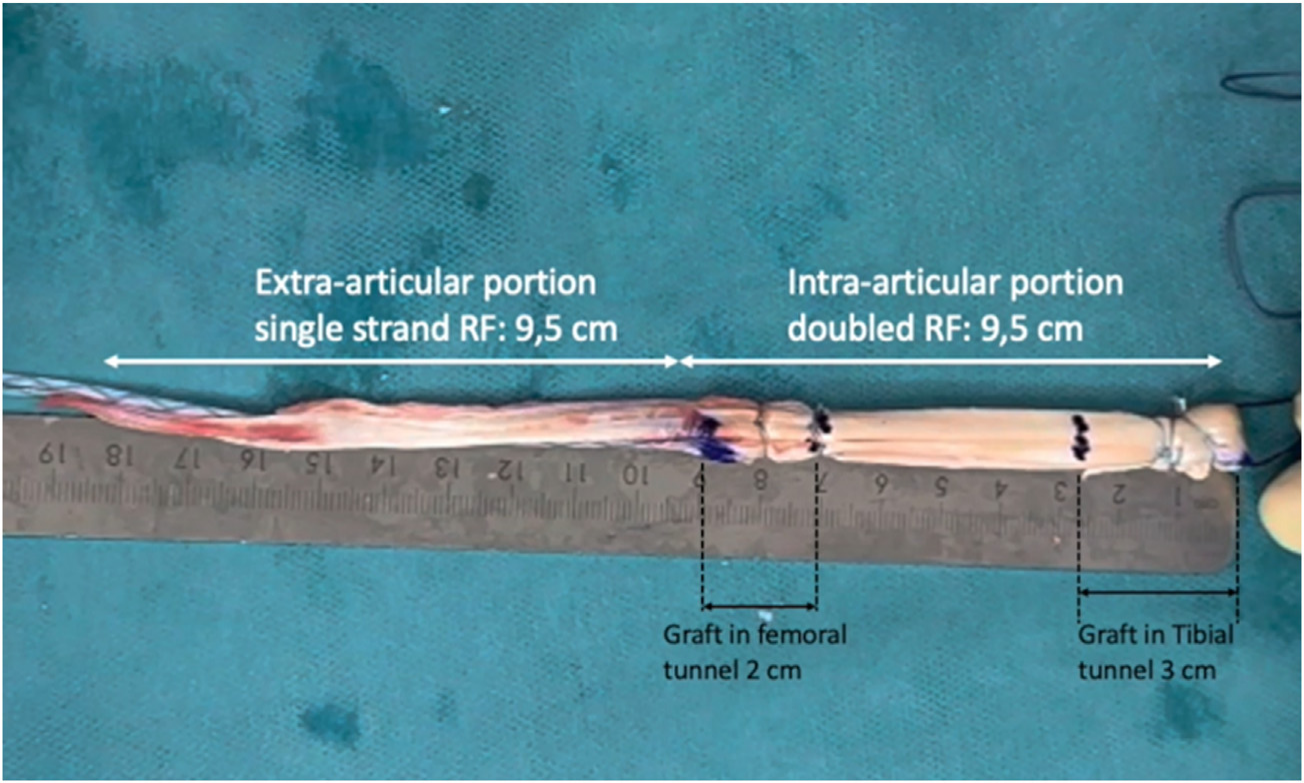
Intraoperative photograph showing the prepared RF graft. The RF tendon is divided into 2 parts: the intra-articular portion, which is folded and sutured using 2 nonabsorbable sutures to create a double-stranded anterior cruciate ligament graft matching the previously measured distance, and the extra-articular portion, which is used to reconstruct the double-stranded anterolateral ligament. (RF, rectus femoris.)

The distance from the tibial tunnel entry point to the femoral tunnel exit point is measured using a suture passed intra-articularly through a looped suture (Video 1). This distance represents the length of the double-stranded intra-articular part of the RF graft. The RF tendon is divided into 2 parts: the intra-articular portion, which is folded and sutured using 2 nonabsorbable sutures to create a double-stranded ACL graft matching the previously measured distance, and the extra-articular portion, which is used to reconstruct the double-stranded ALL (Fig 7).

### RF Graft Passage and Fixation

The intra-articular portion of the prepared graft is passed through the femoral tunnel into the tibial tunnel under direct arthroscopic visualization via the anterolateral portal using a looped suture. The graft is then fixed in both tunnels with 2 interference screws while the knee is maintained in a reduced position.

**Fig 8:**
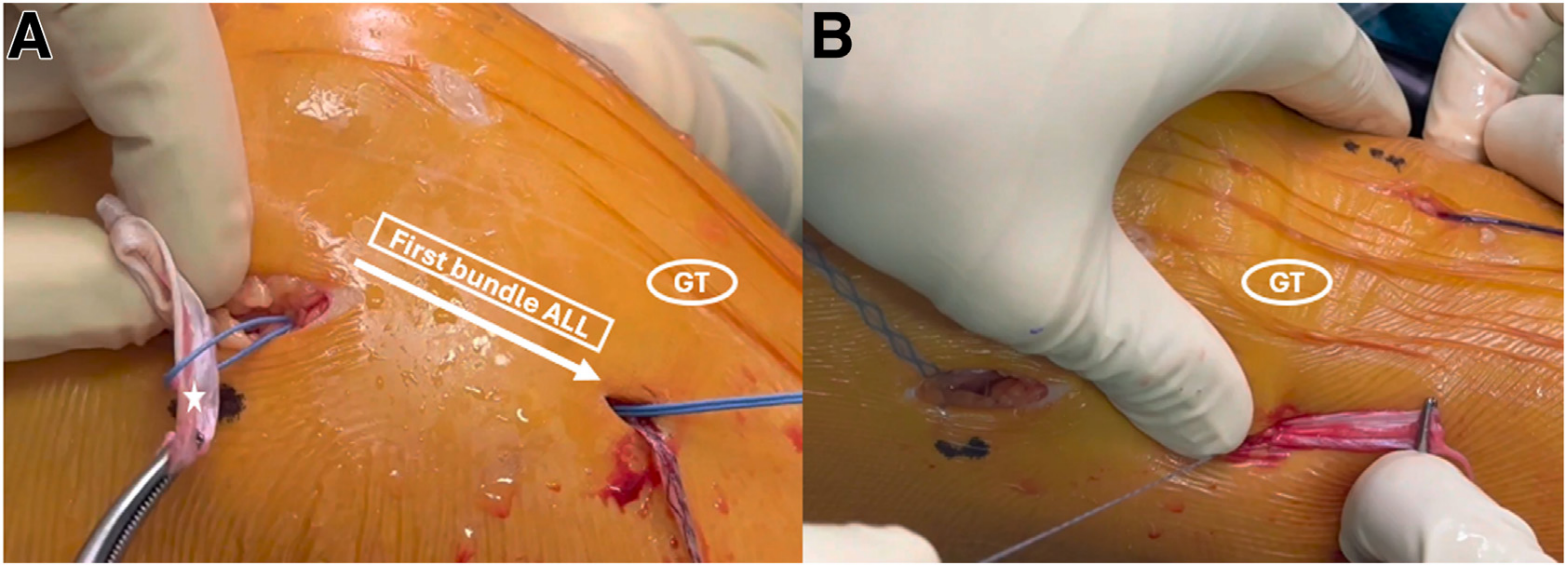
Intraoperative photograph of the left knee, showing the passage of the first bundle of the anterolateral ligament (ALL) under the fascia lata (A) and tibial anchor fixation (B) while maintaining the knee joint in full extension and neutral rotation. The star shows the extra-articular part of the rectus femoris graft. (GT, Gerdy tubercule.)

**Fig 9:**
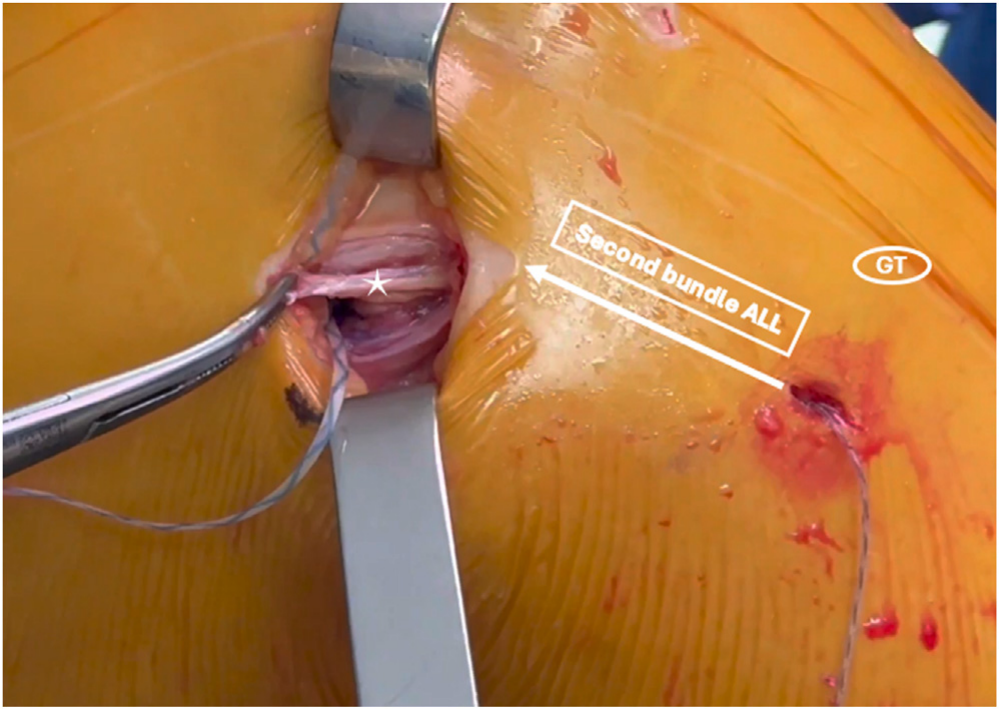
Intraoperative photograph of the left knee showing the passage of the second bundle of the ALL under the fascia lata and its fixation to itself using a No. 2 nonabsorbable suture. The star shows the second bundle of the ALL (ALL, anterolateral ligament; GT, Gerdy tubercule.)

The extra-articular portion of the graft, representing the ALL, is passed under the fascia lata using a looped suture and secured with the FiberTak anchor at the previously prepared tibial insertion (Fig 8). Then, the second strand of the ALL is passed again under the fascia lata and sutured to itself in full knee extension with a No. 2 nonabsorbable suture, which had been previously placed through the loop of the intra-articular part of the RF graft (Fig 9).

### Postoperative Protocol

The postoperative protocol involves immediate weight-bearing with crutches and early progressive range-of-motion exercises to restore full knee extension and improve the quadriceps muscle contraction. Return to sports is allowed at 6 months, starting with nonpivoting activities and progressing to pivoting sports 9 months postoperatively.

## Discussion

This Technical Note describes a surgical technique for ACL and ALL reconstruction using a single RF tendon autograft. A small skin incision is performed for RF tendon harvesting to obtain a graft of sufficient length and diameter for combined ACL and ALL reconstruction, aiming to reduce the risk of graft re-rupture^1^^,^^2^ and minimize harvesting associated complications such as anterior knee pain and sensory loss.^10^ Furthermore, the use of the RF graft for ACL reconstruction in the presence of an associated medial collateral ligament injury is recommended to preserve hamstrings tendon, ensuring optimal valgus stability of the knee joint.^11^

Anatomical double-bundle ALL reconstruction also is performed without the need for a second graft or the creation of additional femoral and tibial tunnels, thereby preventing tunnel convergence.^12^ The use of the FiberTak anchor for ALL tibial fixation helps preserve bone stock^13^ and promotes good ALL graft-bone integration.^14^ In addition, it conserves tendon length for double-bundle ALL reconstruction.

The main risk associated with this technique is postoperative quadriceps muscle weakness, which can be minimized by harvesting only the superficial layer of the RF tendon and avoiding capsular injury. Moreover, the interference should not protrude beyond the femoral cortex to avoid postoperative knee pain related to friction with fascia lata friction and careful attention is required during anchor insertion to prevent anchor breakage.

In our perspective, we believe that our technique provides a valuable alternative for anatomic ACL and double-bundle ALL reconstruction using a single RF autograft. In addition, it’s an effective solution for revision ACL surgeries as a substitute for allografts and in multiligament knee injuries, where preserving the hamstrings is crucial for maintaining optimal valgus stability of the knee joint.

## Disclosures

The authors declare the following financial interests/personal relationships which may be considered as potential competing interests: E.C. reports consulting or advisory with Arthrex. All other authors (V.S-C., A.A., D.M., R.P.) declare that they have no known competing financial interests or personal relationships that could have appeared to influence the work reported in this paper.

## Supplementary Data

Video 1 This instructional video demonstrates the Quad 2.0 technique for anatomical left knee joint anterior cruciate ligament (ACL) and double-bundle anterolateral ligament (ALL) reconstruction. The patient is placed in a supine position with a tourniquet applied to the operative thigh. The superficial rectus femoris tendon is meticulously dissected from the other quadriceps tendons and then harvested to obtain a graft of sufficient length and diameter. The graft is then prepared into 2 parts: an intra-articular portion representing the ACL graft and an extra-articular portion representing the ALL graft. Subsequently, the femoral tunnel entry point is identified posterior and proximal to the lateral epicondyle, followed by the creation of the femoral tunnel using the standard outside-in technique. The tibial tunnel is created using the standard outside-in technique and the graft is passed from the femur to the tibia and fixed with 2 interference screws. The extra-articular part of the graft is passed under the fascia Lata in two directions and fixed to the tibia using the knotless FiberTak soft anchor, allowing for double-bundle ALL reconstruction. The goal of this procedure is to provide an autograft solution as a substitute for allografts in revision ACL surgeries, as well as in multi-ligament knee injuries.
